# Mixture distributions in multi-state modelling: Some considerations in a study of psoriatic arthritis

**DOI:** 10.1002/sim.5529

**Published:** 2012-07-26

**Authors:** Aidan G O'Keeffe, Brian DM Tom, Vernon T Farewell

**Affiliations:** MRC Biostatistics UnitCambridge, U.K.

**Keywords:** multi-state model, mover–stayer model, random effects, psoriatic arthritis

## Abstract

In many studies, interest lies in determining whether members of the study population will undergo a particular event of interest. Such scenarios are often termed ‘mover–stayer’ scenarios, and interest lies in modelling two sub-populations of ‘movers’ (those who have a propensity to undergo the event of interest) and ‘stayers’ (those who do not). In general, mover–stayer scenarios within data sets are accounted for through the use of mixture distributions, and in this paper, we investigate the use of various random effects distributions for this purpose. Using data from the University of Toronto psoriatic arthritis clinic, we present a multi-state model to describe the progression of clinical damage in hand joints of patients with psoriatic arthritis. We consider the use of mover–stayer gamma, inverse Gaussian and compound Poisson distributions to account for both the correlation amongst joint locations and the possible mover–stayer situation with regard to clinical hand joint damage. We compare the fits obtained from these models and discuss the extent to which a mover–stayer scenario exists in these data. Furthermore, we fit a mover–stayer model that allows a dependence of the probability of a patient being a stayer on a patient-level explanatory variable.

## 1 Introduction

Where data are collected on several individuals in a population over time, it is often of interest to fit models that describe the probability of the occurrence of a particular event in time. Such data often occur in the form of a survival study where inference concerns estimating the probability of ‘death’ or ‘failure’ and, clearly, the choice of model is important to ensure an accurate description of the data. Mixture models are often used in survival studies for situations where individuals possess different propensities to undergo the event of interest or where the event of interest may occur owing to different causes for different individuals. Mixture models typically involve a survival distribution that is composed of a mixture of density functions occurring in different proportions, where each density function may represent a different cause or risk of failure 1. The idea of mixture probabilities in such models can be used for studies where interest lies in predicting the proportion of patients who recover or are ‘cured’ from a particular disease and will not experience death from the disease in question. Such models are often known as ‘long-term survival models’ (where the ‘long-term survivors’ are those who will not experience death from the disease) or ‘cure rate models’ (where a cured fraction of patients will not die owing to the disease). Many researchers have explored these models, including Farewell 2,3 who fitted fully parametric cure rate models, which led to a further work 1986. Kuk and Chen 5 developed the cure rate model further, employing a Cox proportional hazards model rather than a fully parametric model. In these works, and in many others, a common problem is choosing the most appropriate form of mixture distribution to best describe the disease or failure process under observation. We attempt to consider this problem more closely in this paper.

A multi-state model is an extension of a survival model to processes that change over time but that may occupy a finite number of discrete states 6–9. In many diseases, it may be suspected that there are some members of the population for whom disease progression is unlikely to occur. In a multi-state setting, such individuals are known as ‘stayers’ (i.e. those patients who ‘stay’ in the same disease state as time passes), whereas those who progress are known as ‘movers’. Multi-state models to account for such situations are known generally as ‘mover–stayer’ models and have been used in many studies (e.g. 10–12). Often, a patient-specific random effect, in the form of a finite mixture distribution, is used in a mover–stayer multi-state model to account for the sub-populations of movers and stayers. A random effect provides a measure of the propensity of a patient to progress through the states of the model and can also account for unobserved heterogeneity amongst patients. Usually, the proportion of patients who are stayers is represented by a point mass at zero in the random effects distribution, and this is the case for all mover–stayer random effects distributions that are considered in this paper. Such models are analogous to frailty models in survival and event history analysis (e.g. 13,14), although frailty models in survival data are not generally fitted with the aim of investigating finite sub-populations within a data set. Often, choosing a mover–stayer random effects distribution to use in a multi-state model can be problematic. Different mover–stayer random effects distributions may yield widely differing inferences, and it can be difficult to decide which, if any, mover–stayer model provides the best description of a particular data set.

**Figure 1:**
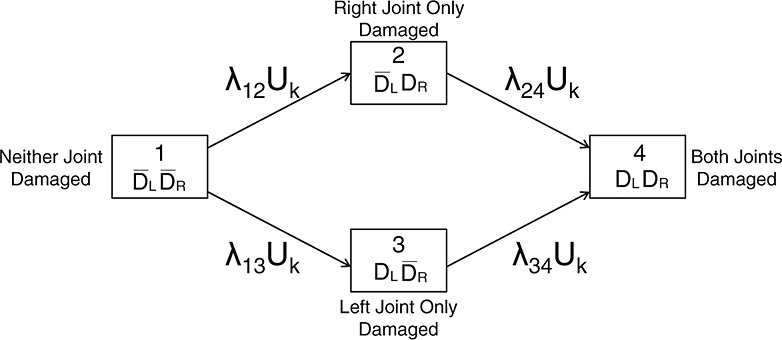
Diagram of the multi-state model for damage at a joint location, with random effect.

The work in this paper is motivated by a reasonably complex multi-state model to describe the progression of clinical joint damage in psoriatic arthritis (PsA) patients. We consider a four-state multi-state model (shown diagrammatically in Figure 1) for the clinical damage process in each hand joint location, of which there are 14 for each patient. We combine information on clinical damage from the left and right joints at a particular location, resulting in the following four states:

1. State 1: Damage in neither joint;
2. State 2: Damage in the left hand joint only;
3. State 3: Damage in the right hand joint only;
4. State 4: Damage in both the left and right joints.

We fit the four-state model (Figure 1) to each joint location. Because there is likely to be correlation amongst the joint locations within each individual, we use a patient-specific random effect (denoted by *U*_*k*_ for the *k*th patient) to account for this. In addition, we may also use the random effect to determine whether the patient is a mover or a stayer, taking the value *U*_*k*_ = 0 for stayers and *U*_*k*_ > 0 otherwise. Clearly, there are many different choices of random effects distribution, and we aim to investigate possible mover–stayer random effects distributions for this purpose. The random effects distributions considered are a mover–stayer gamma, a mover–stayer inverse Gaussian and a compound Poisson (CP). We took the data from a large cohort of PsA patients under observation at the University of Toronto. Our main aims in this work are to consider features of mover–stayer multi-state models, where several multi-state models are clustered within a subject. This includes a consideration of methods that can be used for model fitting, how the analyses resulting from such models may be interpreted and some discussion on the problem of selecting a suitable mover–stayer random effects model.

## 2 The data and modelling techniques

### 2.1 Psoriatic arthritis and the University of Toronto study

Psoriatic arthritis is an inflammatory arthritis associated with the skin disease psoriasis. Disease progression in PsA is usually reflected in the accumulation and severity of damaged joints, which can be evaluated clinically or by using radiographic imaging. In this paper, ‘damage’ refers to clinical damage, rather than radiological damage, to a joint. Extensive data from the University of Toronto PsA clinic were first collected in 1978, and since then, over 1000 patients have been followed up longitudinally, with clinic visits scheduled to be 6–12 months apart. At each clinic visit, several measurements are taken, according to a defined protocol, including the following: results from a physical examination, a complete history, routine blood and urine tests, along with biennial X-rays. As a result, a detailed and accurate record of the state of each joint at each clinic visit is known, which may be used to perform multi-state modelling of disease progression at the individual joint location.

### 2.2 A four-state multi-state model for damage

Throughout this work, we consider a four-state multi-state model for clinical hand joint damage (Figure 1). We generally assume the damage process to be irreversible, which implies that transitions are not permitted from states of damage to states of non-damage. Moreover, we assume that the damage processes in the left and right joints cannot change state (i.e. move from a state of no damage to damage) simultaneously, which prohibits a transition from state 1 to state 4. This is not particularly restrictive, because damage is assumed to occur in continuous time.

A typical multi-state model assumes that the movement within a state space (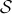) of *K* discrete states (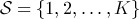) is governed by an underlying stochastic process 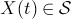. The transitions amongst the states of a multi-state model are governed by a *K* × *K* matrix of transition intensities, Λ(*t* | 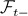), where the (*r*,*s*) element of Λ(*t* | 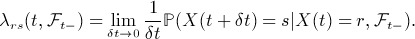) represents the instantaneous rate of transition from state *r* to state *s* ( (*r*,*s*) ∈ {1, …, *K*} × {1, …, *K*}) and 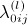 denotes the history of the process *X* up to a time immediately prior to *t*. That is,

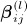


In PsA, there is much clinical interest in the relationship between disease activity and joint damage. Disease activity occurs in the form of tenderness and/or effusion (swelling) at a joint, where effusion represents a more severe level of activity than tenderness. Many clinicians believe that there may be a causal link between disease activity and clinical joint damage. To account for this belief, we allow explanatory variables measuring joint activity to act on the transition intensities of the multi-state model (Figure 1) when fitting models. Suppose that the baseline transition intensity from state *i* to state *j* at joint location *l* is written as 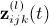 with 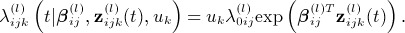, a vector of regression parameters indicating the effects of a vector of individual-specific explanatory variables, 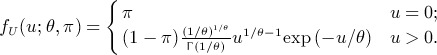, which act upon the state *i* to state *j* baseline transition intensity at time *t*. Assuming that *u*_*k*_ represents the realisation of the random effect, *U*_*k*_, for the *k*th patient, the form of the state *i* to state *j* transition intensity for the *k*th patient at joint location *l* and time *t* is given by

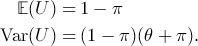
(1)
We estimated the explanatory variable effects, baseline intensities and random effects distribution parameters (together with associated standard errors) by using maximum likelihood methods. We calculated the log-likelihood for the model by using the statistical program R 15 and performed maximisation of the log-likelihood function by using the ‘Broyden–Fletcher–Goldfarb–Shanno’ (BFGS) optimisation method 1970, implemented using the optim command. We provide the details in Appendix A.

## 3 Random effects distributions

The principal aim in this work was to compare the use of different random effects distributions to account for both the inherent correlation amongst joint locations and the possibility of a mover–stayer scenario with regard to hand joint damage in the Toronto PsA cohort. Previous work 17 used a (non-mover-stayer) gamma distribution, with unit mean, as the random effects distribution for the same four-state multi-state model (Figure 1) fitted to the same data set. This work concentrated on using multi-state modelling to investigate the possibility of a causal relationship between activity and damage in the hand joints of patients from the Toronto PsA cohort. In our current work, we compare the multi-state model fitted in 17 with that fitted using mover–stayer random effects distributions, which will be described later. In addition, we note that an individual is defined to be a stayer if and only if each one of his or her hand joints does not have a propensity to develop clinical damage. One could argue that an individual may have a propensity to develop clinical damage in some joints but not others; however, this would lead to a more complex mover–stayer model that would be very difficult to fit computationally. Also, this would represent a very different clinical question and perhaps not an intuitive one, given that the Toronto PsA data contain so many individuals who do not develop clinical damage in any one of the hand joints during their time in the study. In addition, for patients who are observed to develop damage during their time in the study, damage occurs at a fast rate. Out of the 148 observed movers, 100 patients exhibited damage in multiple joints during their time in the study. Of these 100 patients, 68 had damage in multiple joints at the visit when damage was first observed to occur (with mean time until first observed damage of 7.32 years), and 32 patients had damage in one joint only at the visit where damage was first observed to occur (with mean time until first observed damage of 7.03 years). For the 48 patients who exhibited damage in only one joint during their time in the clinic, the mean time until first observed damage was 7.03 years. We note that, of these three groups, the group of patients who exhibit only one damaged joint during their time in the clinic have the shortest mean follow-up time of 10.92 years compared with 14.36 years for patients who exhibit initial damage at multiple joints and 17.39 years for patients who exhibit damage in one joint only before developing further damage while under observation.

### 3.1 Mover–stayer gamma distribution

For the mover–stayer gamma distribution, the random effects have a value of zero for the stayers and follow a Gamma(1 / *θ*,1 / *θ*) distribution for those who are movers. The parameter *π* denotes the probability that an individual is a stayer, that is, *π* = P(Stayer) and the probability density function for the distribution is given by

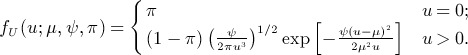
(2)
The expectation and variance of this distribution are

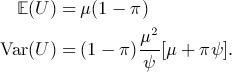
(3)

### 3.2 Mover–stayer inverse Gaussian distribution

For the mover–stayer inverse Gaussian distribution, the random effects have a value of zero for the stayers and follow an inverse Gaussian distribution, with mean *μ* and shape parameter *ψ*, for the movers. The form of the probability density function for *U*, where *π* = P(Stayer) is given by

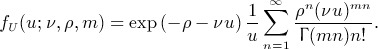
(4)
The expectation and variance for this distribution are

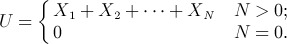


### 3.3 The power variance function family of distributions

A more general distribution family that accounts for sub-populations of movers and stayers is the power variance function (PVF) family of distributions. The PVF family is a three-parameter family first introduced by Hougaard 18 and extended in 19. The PVF family, having parameters *ν*, *ρ* and *m*, is described as those distributions where if *U* is a PVF random variable, then the density of *U* is given by

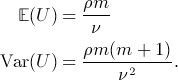
(5)
Here, *ν* > 0, *m* > − 1 and *mρ* > 0. This form of the distribution is defined in 20, p. 238, where the authors note that the PVF family includes both the gamma distribution (obtained where *ρ* → ∞ and *m* → 0 such that *mρ* → *ν*) and the inverse Gaussian distribution (obtained where *m* = − 1 / 2 and *ρ* < 0). Where *m* > 0, a CP distribution is obtained, and it is a form of CP distribution that will be used as the third mover–stayer random effects distribution in the four-state multi-state model (Figure 1).

If *U* denotes a CP patient-specific random effect, then *U* may be written as the sum of *N* gamma-distributed random variables where the number of summands, *N*, has a Poisson distribution with expectation *ρ*. That is,

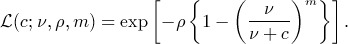
(6)
Here, each *X*_*i*_ ∼ Gamma(*m*,*ν*)(*i* = 1, …, *N*), and this implies that *U* | *N* = *n* > 0 ∼ Gamma(*mn*,*ν*). The CP distribution consists of a positive probability mass at zero and a density along the positive real line. The point mass at zero has probability exp( − *ρ*), implying that P(Stayer) = exp( − *ρ*). The expectation and variance of *U* are

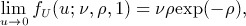

The Laplace transform of the CP distribution is given by

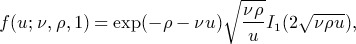


If we consider the behaviour of the density (Equation 7) as *u* → 0, then we see that

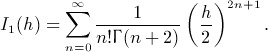
(7)
and we provide a formal proof of this result in Appendix B. Several authors have used the CP distribution as a frailty distribution in survival studies 21–23. Recent work on a bowel cancer study 24 used a CP random effect in a progressive three-state ‘chain-like’ multi-state model. In these situations (frailty models and progressive chain-like multi-state models), the CP model can be fitted easily owing to its convenient Laplace transform and to the fact that in the survival models, there exists only one possible transition. In general, these works have not considered a series of clustered multi-state models within an individual, as is the case for hand joint damage in the University of Toronto PsA cohort.

A special case of the CP distribution is that where *m* = 1, and the probability density function (Equation 7) exhibits the following closed form:

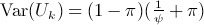
(8)
where *I*_1_(*h*) is a modified Bessel function of the first kind. That is,

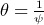
(9)
The closed form is useful when attempting to fit a CP random effects distribution to correlated multi-state models. This is because the Laplace transform cannot be used to fit the model for all patients, as the different joint locations in a patient may have differing transition paths through the states of the model. Numerical integration is required to fit the model, and a closed distributional form is clearly more desirable for this purpose. We note that the chosen CP-PVF distribution is still flexible, because *ρ* and *ν* remain free to vary. We will refer to this distribution henceforth as the CP-PVF distribution.

## 4 Mover–stayer models in the Toronto psoriatic arthritis study

As discussed previously, much of the analysis of the Toronto PsA cohort has been to investigate the relationship between activity and damage in the hand joints. The remainder of this paper concentrates on a comparison of the three mover–stayer distributions, described earlier, for this purpose.

### 4.1 Toronto psoriatic arthritis data

We considered data from 510 patients of the Toronto PsA cohort who were chosen because they exhibited no clinical damage in any of the hand joints at entry to the clinic. Of these 510 patients, there are 362 (approximately 71%) who had no damage in any of the hand joints during their time in the study. For patients who were observed to be always damage free throughout their time in the study, their mean age at clinic entry was 41.5 years (standard deviation = 12.7 years). The earliest entry to the clinic was in January 1978 with the latest clinic entry, prior to January 2007, occurring in July 2006. We note that approximately 69% of the patients observed to be always damage free had entered the study before 2000. Their mean duration in the study was 6.9 years (ranging from 3 months to 28.4 years), and their median number of clinic visits was 6 (ranging from 2 to 46).

### 4.2 Model fitting

We used each of the three mover–stayer random effects as the patient-specific random effect in the four-state multi-state model for hand joint damage (Figure 1). The mover–stayer gamma distribution used is the same as that used in Equation 3. The mover–stayer inverse Gaussian distribution used was chosen with parameter *μ* = 1 and the *ψ* parameter free to vary. We note that where *μ* = 1, the expectation of the mover–stayer inverse Gaussian becomes *E*(*U*_*k*_) = 1 − *π*, and the variance is given by 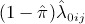. These first two moments are identical to those for the mover–stayer gamma distribution (Equation 4), with the change in parameter 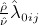, although the shape of the distribution is not the same.

The CP-PVF model chosen has probability density function given by Equation 12, with a point mass of size exp( − *ρ*) at zero, such that P(Stayer) = exp( − *ρ*). For the CP-PVF model, we note that the expectation of the random effect is given by *ρ* / *ν*. As such, setting the CP-PVF distribution to have unit expectation would have forced the undesirable constraint of *ρ* = *ν*, resulting in a less flexible CP-PVF random effects distribution. Instead, we set the baseline state 1 to state 2 transition intensity, *λ*_012_, to be equal to one. Hence, we can consider the estimate of *ρ* / *ν*, obtained using maximum likelihood estimation, to be an estimate for the state 1 to state 2 baseline transition intensity. We present the results from fitting the mover–stayer gamma, mover–stayer inverse Gaussian and CP-PVF distributions as patient-specific random effects in the four-state model (Figure 1). We chose explanatory variables concerning the activity level at a joint to act on the transitions of the model by using the relationship shown in Equation 2. The three binary variables used are tenderness only ( 1 = tenderness only in the joint and 0 = otherwise), effusion with or without tenderness ( 1 = effusion with or without tenderness in the joint and 0 = otherwise) and past activity ( 1 = joint has been active in the past and 0 = otherwise). The explanatory variable to describe present activity (tenderness or effusion) in the opposite joint of a pair to the joint undergoing a transition to damage is a binary variable such that 1 = joint active presently and 0 = joint not active presently.

### 4.3 Results

Table 1 shows the results of fitting the multi-state model (Figure 1) to the Toronto PsA data, using the three mover–stayer random effects distributions, described in Section 3. The table also shows results where a (non-mover-stayer) gamma distribution was used as a random effects distribution 17 to compare the explanatory variable effects. We note that for each mover–stayer distribution, the estimate of P(Stayer) was constrained to be the same for all individuals although this will be relaxed later. A ‘transitive’ joint indicates a joint that may experience a transition to damage at a particular location (i.e. a joint for which the transition rate is being modelled), and an ‘opposite’ joint refers to the same joint on the opposite hand at a particular location.

**Table 1:** Intensity ratio estimates together with associated 95% confidence intervals fitted to the model incorporating activity and damage to each individual joint pair of the left and right hands by using mover-stayer (M-S) random effects.

Intensity ratio				
No previous damage in either joint				
Effect on transition to damage	Gamma	M-S gamma	M-S inverse Gaussian	CP-PVF
Tenderness in the transitive joint	2.76 (2.06, 3.70)	2.75 (2.05, 3.69)	2.76 (2.19, 3.46)	2.74 (2.05, 3.66)
Effusion in the transitive joint	4.47 (3.38, 5.90)	4.46 (3.38, 5.88)	4.51 (3.92, 5.19)	4.32 (3.28, 5.68)
Activity in the opposite joint	1.18 (0.90, 1.55)	1.18 (0.90, 1.56)	1.17 (0.94, 1.46)	1.20 (0.92, 1.57)
Transitive joint active in the past	2.14 (1.68, 2.71)	2.14 (1.68, 2.73)	2.14 (1.88, 2.42)	2.07 (1.64, 2.62)
Opposite joint active in the past	1.10 (0.86, 1.41)	1.10 (0.86, 1.41)	1.10 (0.97, 1.25)	1.07 (0.84, 1.37)
Opposite joint damaged				
Effect on transition to damage	Gamma	M-S gamma	M-S inverse Gaussian	CP-PVF
Tenderness in the transitive joint	2.24 (1.51, 3.32)	2.26 (1.53, 3.35)	2.23 (1.91, 2.59)	2.29 (1.55, 3.38)
Effusion in the transitive joint	2.19 (1.40, 3.41)	2.21 (1.42, 3.44)	2.18 (1.87, 2.54)	2.27 (1.46, 3.53)
Transitive joint active in the past	1.37 (1.00, 1.86)	1.37 (1.01, 1.86)	1.38 (1.20, 1.59)	1.35 (1.00, 1.84)
Baseline intensities				
Parameter ( × 10^− 2^)	Gamma	M-S gamma	M-S inverse Gaussian *	CP-PVF *
*λ*_012_	0.28 (0.21, 0.36)	0.29 (0.22, 0.38)	0.28 (0.16, 0.49)	0.26 (0.20, 0.34)
*λ*_013_	0.27 (0.21, 0.34)	0.28 (0.21, 0.37)	0.27 (0.15, 0.48)	0.25 (0.19, 0.32)
*λ*_024_	2.15 (1.49, 3.10)	2.27 (1.57, 3.30)	2.11 (1.14, 3.89)	1.95 (1.67, 3.29)
*λ*_034_	2.34 (1.58, 3.47)	2.43 (1.61, 3.66)	2.44 (1.09, 5.46)	1.94 (1.54, 3.32)
Random effect parameters				
Parameter	Gamma	M-S gamma	M-S inverse Gaussian	CP-PVF
*θ*	3.81 (2.98, 4.88)	3.57 (2.65, 4.81)		
*ψ*			0.33 (0.29, 0.37)	
*ν*				176.43 (138.06, 225.48)
*ρ*				0.46 (0.39, 0.55)
Estimate of P(Stayer)		M-S gamma	M-S inverse Gaussian	CP-PVF
		0.042 (0.001, 0.797)	0.334 (0.255, 0.437)	0.631 (0.579, 0.679)

For reference, we also show in this table results using the gamma distribution from 17.

* We calculated these estimates and confidence intervals as estimated *expected values* for each distribution. That is, 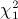 for the mover–stayer (M-S) inverse Gaussian distribution and 
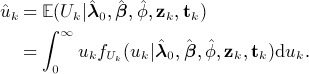
 for the compound Poisson power variance function (CP-PVF) distribution.

The results in Table 1 indicate that the effects of tenderness, effusion and activity (past and current) show a similar effects pattern across the different choices of mover–stayer distribution. The effects pattern shows that where neither joint at a particular location is damaged, tenderness, effusion and past activity in the transitive joint all have large, significant effects on the transition to damage. Conversely, both current activity (tenderness or effusion) and past activity in an opposite joint do not have significant effects on the transition to damage. Where an opposite joint at a particular location is already damaged, tenderness and effusion have significant effects on the transition to damage although we no longer observe a stronger effect for effusion in the transitive joint compared with the effect of tenderness only as was the case where neither joint at a particular location exhibited damage. Past activity has a marginally significant effect on the transition to damage, and the effect is generally smaller than that seen where neither joint in a pair is previously damaged. The baseline transition intensity estimates are generally similar across the different random effects models.

**Figure 2:**
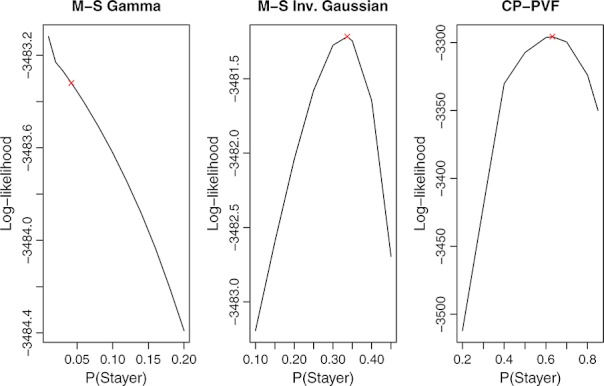
Plots of the profile log-likelihood for various values of P(Stayer). The ‘ × ’ indicates the point to which the numerical optimisation procedure converged.

However, the three mover–stayer distributions have resulted in widely varying estimates of P(Stayer) = *π*, with the mover–stayer gamma, mover–stayer inverse Gaussian and CP-PVF distributions producing estimates (with associated 95% confidence intervals in parentheses) of 0.042 (0.001, 0.797), 0.334 (0.255, 0.437) and 0.631 (0.579, 0.679), respectively. The estimate of *π* close to zero together with the associated wide confidence interval seen for the mover–stayer gamma distribution may suggest that there is little evidence, or great uncertainty, to determine whether the population contains separate sub-populations of movers and stayers, from this model. To investigate the differences in these estimates further, we produced plots of the profile log-likelihood for various values of *π*, along with empirical Bayes estimates of the random effects for each distributional choice. Figure 2 shows plots of the profile log-likelihood calculated for each mover–stayer distribution for various values of P(Stayer).

When a statistical model is specified for a data set, the likelihood function is generally used to provide information for the estimation of model parameters. For the mover–stayer models considered, the proportion of patients who remain damage free is, in each case, governed by one model parameter only (*π* in the mover–stayer gamma and mover–stayer inverse Gaussian distributions and *ρ* in the CP-PVF distribution). An examination of the shape of the profile log-likelihood, in each case, for these parameters allows an assessment of the identifiability of the proportion of patients who remain always damage free. A single peak in the profile log-likelihood plot for these parameters would suggest that an estimate for P(Stayer) is identifiable. Conversely, a relatively flat profile log-likelihood shape would suggest difficulty in identifying a maximum likelihood estimate for P(Stayer).

**Figure 3:**
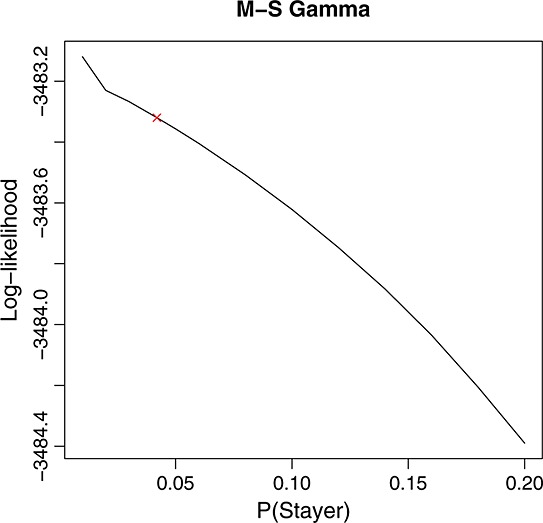
Plot of the profile log-likelihood for various values of P(Stayer), for values of π close to that at which the numerical optimisation procedure converged for the mover-stayer gamma model. The × indicates the point to which the numerical optimisation procedure converged.

Figures 2 and 3 show plots of the profile log-likelihood for various values of P(Stayer) in each of the three mover–stayer models that were considered. The shape of the profile log-likelihood for *π* for the mover–stayer gamma model suggests that the profile log-likelihood increases as *π* approaches zero. The obtained estimate from the numerical optimisation procedure clearly does not occur at the point where the likelihood is maximised, implying that the optimisation process has not been successful at obtaining a maximum likelihood estimate for *π*. In essence, the shape of the profile log-likelihood for *π* appears to suggest that the maximum likelihood estimate for *π* would be effectively equal to zero. Clearly, the shape of the profile log-likelihood for *π* for this model is not concave, so it is unsurprising that the optimisation process has not attained the maximum likelihood estimate for *π*. However, it would appear that the other model parameter estimates (and associated asymptotic confidence intervals) appear reasonable and identifiable when compared with those obtained using a non-mover-stayer gamma random effects distribution. Because the maximum likelihood estimate was not attained, it is not possible to obtain an asymptotic 95% confidence interval for *π* using standard methods. As an alternative, we calculated a 95% likelihood ratio interval for *π* as (0.00, 0.30). This interval represents the values of *π*_0_ for which we are unable to reject the null hypothesis *π* = *π*_0_ in a likelihood ratio test. Given these results, the addition of a mover–stayer component to the gamma distribution does not appear to be necessary.

For the mover–stayer inverse Gaussian model, a peak is seen clearly in the plot of the profile likelihood for *π* (Figure 2). This suggests that the maximum likelihood estimate for *π* is identifiable for this model. We calculated this estimate for *π* as 0.334 with 95% Wald confidence interval (0.225, 0.437). This confidence interval does not lie close to zero, thereby implying that there is some evidence to support a mover–stayer scenario with regard to hand joint damage. We performed a generalised likelihood ratio test between this model and that where a non-mover-stayer inverse Gaussian distribution was used for the random effects. Because this is a test of the null hypothesis that *π* = 0, we compared the test statistic with a 50:50 mixture of a 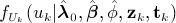 distribution and a point mass at zero. This yielded a test statistic of 6.31 with a corresponding *p*-value of 0.006, suggesting that the inclusion of a term to account for P(Stayer) is necessary for this model. Consequently, this provides evidence to suggest that a mover–stayer scenario may exist within these data, with regard to clinical damage in the hand joints.

The plot of the profile log-likelihood for the CP-PVF distribution shows an obvious peak, and the use of this distribution for the random effects yielded a maximum likelihood estimate of P(Stayer) = 0.631, with a relatively narrow 95% confidence interval of (0.579, 0.679). This suggests that the estimate for P(Stayer) is identifiable from these data under this distributional assumption. The 95% confidence interval does not lie close to zero, and hence, this model provides evidence to support the existence of a mover–stayer scenario with regard to hand joint damage. The CP-PVF distribution does not represent the same type of mixture distribution as the mover–stayer gamma and mover–stayer inverse Gaussian distributions. This is because the mover–stayer gamma and mover–stayer inverse Gaussian distributions had a separate parameter (*π*), which governed the proportion of stayers in the model only. The CP-PVF model contains the parameter *ρ* on which the proportion of stayers and the continuous part of the random effects distribution for the movers both depend.

We considered next empirical Bayes estimates of the random effect, *U*_*k*_, for each patient under each of the chosen random effects distributions (including the original gamma distribution). Suppose that *ϕ* denotes the set of parameters of the random effects distribution. Then the empirical Bayes estimates of *u*_*k*_ are given by

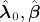

**Figure 4:**
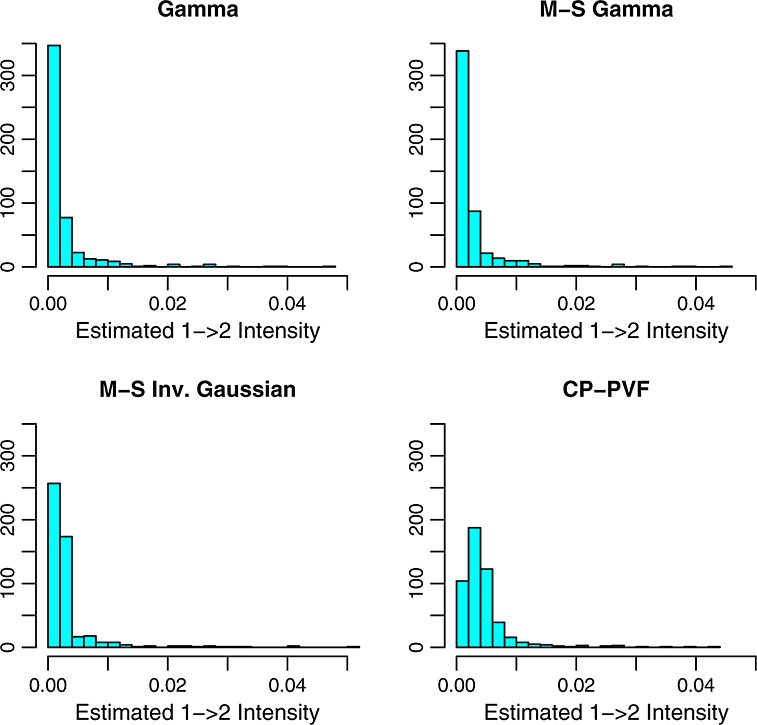
Histograms showing the estimated state 1 to state 2 baseline transition intensities, conditional on the patients being ‘movers’.

**Figure 5:**
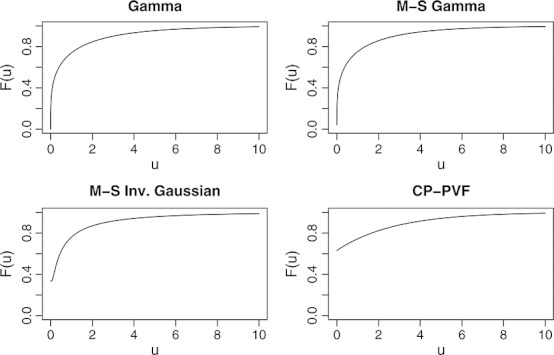
Plots showing the cumulative density functions of each random effect distribution. In each case, the distribution parameters are the maximum likelihood estimates. M-S gamma, M-S inv. Gaussian and CP-PVF distributions have, in each case, been rescaled to have unit mean.

Here, 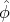 denotes the predictive distribution of *u*_*k*_, given the parameter estimates 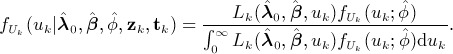 and 
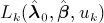
. That is,

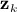

where 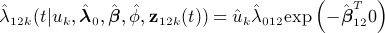 is the model likelihood contribution from the kth patient, having explanatory variables 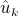 at times **t**_*k*_. The random effects distributions considered have different expected values, and so estimates of *U*_*k*_ for each distribution are not directly comparable. Hence, to compare the random effect distributions, we chose to compare the estimated state 1 to state 2 transition intensities, under the assumption that all explanatory variables have a value of zero, for each patient. The state 1 to state 2 intensity estimate is given by

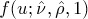

where 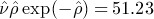 is the empirical Bayes estimate of the random effect. In Figure 4, we show histograms of the estimated state 1 to state 2 baseline intensities, conditional on the patient being classed as a mover. Figure 5 shows plots of the random effects distribution cumulative density functions (CDFs) for each distribution type, whose parameters are estimated in Table 1. For the gamma and mover–stayer gamma random effects distributions, the histograms of empirical Bayes estimates of the state 1 to state 2 transition intensities (Figure 4) show that most random effect values lie near to zero. Conversely, the histogram for the CP-PVF distribution shows estimated values for the state 1 to state 2 transition intensities that are less skewed towards zero. The CDFs (Figure 5) show that the CDF shapes are very similar for the mover–stayer gamma and non-mover-stayer gamma models. The choice of a gamma distribution for the random effects allows the random effects distribution to have a substantial mass near to zero, which may make the identification of a sub-population of stayers difficult. This idea is supported by the shape of the profile log-likelihood (Figure 2) and the confidence interval for the estimate of *π* seen when a mover–stayer gamma random effects distribution was assumed. The inverse Gaussian CDF also has a similar shape although the probability density function for the continuous part of this distribution (Equation 5) has a limit of zero as *u* → 0, which may be why the estimation of P(Stayer) is less problematic for this random effects distribution. In contrast, the CP-PVF CDF increases at a slower rate than that for the other distributions despite the fact that the continuous part of this CDF begins at P(Stayer) = 0.63. This suggests that the random effect values for the population of movers are less skewed towards zero for this random effects distribution, which is consistent with the shape of the empirical Bayes estimates histogram for the state 1 to state 2 baseline intensities in Figure 4.

**Figure 6:**
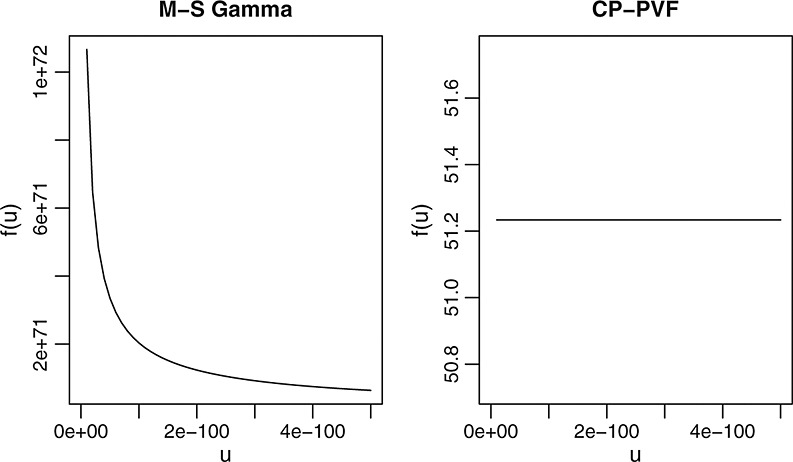
Plots showing the probability density functions of the mover–stayer gamma and CP-PVF random effect distributions for values of *u* close to zero.

The probability density function shape for the mover–stayer inverse Gaussian random effects distribution clearly does not asymptote towards ∞ as *u* → 0. However, the histogram for the empirical Bayes estimates of the state 1 to state 2 transition intensities suggests that values of the random effects tend to lie near zero, although this is less so than where the mover–stayer gamma random effect was used. From the estimation process, we obtained an estimate of P(Stayer) with a reasonable confidence interval, suggesting that the problem of *π* not being estimable is not present when a mover–stayer inverse Gaussian distribution is used for the random effects. The continuous part of the estimated CP-PVF probability density function may appear to asymptote to infinity as *u* → 0. However, Equation 11 indicates that 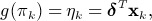 asymptotes to 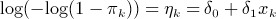 as *u* → 0. This is verified by Figure 6, which shows the probability density function for the mover portions of both the mover–stayer gamma and CP-PVF distributions at values of *u*, which lie very close to zero, and is proved formally in Appendix B. The plots show that the CP-PVF distribution asymptotes towards *f*(*u*) = 51.23 as *u* → 0 whereas that for the mover–stayer gamma distribution takes very large values and is increasing as *u* → 0. This, together with both the shape of the profile log-likelihood for P(Stayer) and the estimate of P(Stayer) exhibiting a relatively narrow 95% confidence interval, implies that estimation of the proportion of damage-free patients under the CP-PVF random effects specification is less problematic than the case of the other mover–stayer random effects distributions considered. It may be that, as mentioned previously, the dependence of both P(Stayer) and the continuous part of the CP-PVF distribution on a common parameter (*ρ*) may result in this less problematic estimation of model parameters, under the assumption of a mover–stayer scenario within the data set.

It is important to note that the time origin used for all patients was the time at clinic entry. Clearly, some patients may have had the disease longer than others at this point, which may be informative about the proportion of patients who are stayers. We considered only those patients who had no clinical damage in all joints at clinic entry so that all patients were either stayers or movers who had not yet progressed, in an effort to make patients more comparable at this point. We recognise that additional controls, such as incorporating explanatory variables measuring the time since diagnosis or other suitable measures of disease duration could be included, if desired.

### 4.4 Choosing the most appropriate model

The application of each of the three random effects distributions to these sets of four-state multi-state models resulted in similar estimates of the baseline transition intensities and explanatory variable effects. This is encouraging from the viewpoint of making robust conclusions with regard to the relationship between the behaviour of the activity and damage processes for these patients in continuous time. In short, we draw the same conclusions about this relationship regardless of the patient-level random effects distribution. The main aim in fitting the mixture models was to assess whether a mover–stayer scenario with regard to hand joint damage exists within these data. Two of the mover–stayer random effects models considered (the mover–stayer inverse Gaussian and CP-PVF) provided some evidence to suggest that a mover–stayer scenario exists with regard to hand joint damage. Conversely, the mover–stayer gamma random effects model provided insufficient evidence in support of the existence of a mover–stayer scenario. It is not possible to assess directly which of these three models represents the best description of the hand joint damage process in the Toronto PsA study, and we cannot know how close estimates of P(Stayer) lie to the (unknown) true value of P(Stayer). Nevertheless, it seems intuitive to examine the features of these fitted models in an effort to make a pragmatic assessment as to which models are the most appropriate for these data.

When a non-mover-stayer gamma model was fitted to these data 17, the shape of the density function was such that the density increased towards ∞ as *u* → 0. This could suggest that, under the assumption that there is no mover–stayer scenario, some members of the population would generally have small random effect values and thus a low rate of progression in the model. Conversely, this may suggest that a mover–stayer scenario could exist but that the presence of a group of stayers has drawn the shape of the random effect distribution towards zero. When the gamma distribution was extended to a mover–stayer gamma distribution, the CDF shape for the movers was almost identical to the shape of that for the non-mover/stayer gamma distribution (Figure 5). This implies that the fitting process for the mover–stayer gamma model results in a large proportion of probability density being concentrated on those movers who progress very slowly. This suggests that it is difficult to distinguish between true stayers and ‘slow-rate movers’ for this data set, which may explain why it was difficult to obtain an estimate of *π* for this model. The other mover–stayer densities that we considered do not asymptote to ∞ as *u* → 0, which may help to limit the occurrence of problems with regard to identifying the proportion of stayers in this data set.

The mover–stayer inverse Gaussian model provided evidence to suggest that a mover–stayer scenario with regard to hand joint damage exists within these data. We obtained this evidence by using a *p*-value from a generalised likelihood ratio test between the non-mover-stayer and mover–stayer inverse Gaussian random effects models and by noting that the asymptotic 95% confidence interval for P(Stayer) indicated a substantial departure from zero. The CP-PVF model also provided evidence in support of the existence of a mover–stayer scenario. Although a generalised likelihood ratio test against a non-mover-stayer model was not possible in this case, the substantial departure of the 95% confidence interval for P(Stayer) from zero implies that a mover–stayer scenario exists. For both models, an examination of the profile log-likelihood for P(Stayer) suggested that the maximum likelihood estimate for P(Stayer) is identifiable, and the shapes of the estimated random effects distributions for movers indicate that both of these models are reasonable for these data. Hence, there appears to be a mover–stayer scenario with regard to hand joint damage. However, although these two random effects models suggest a mover–stayer scenario, they provide different estimates of P(Stayer). There is no definitive method of deciding which of these two models best represents these data. The mover–stayer inverse Gaussian random effects distribution produced a smaller estimate for P(Stayer) with a larger standard error than that where the CP-PVF random effects distribution was used. In addition, we note that the estimate of P(Stayer) of 0.631 obtained using the CP-PVF model lies closer to the empirical estimate for P(Stayer) of 0.71 than the corresponding estimate of 0.334 obtained where a mover–stayer inverse Gaussian random effects distribution was employed. The model where a CP-PVF random effects distribution was used had the largest maximised log-likelihood of the mover–stayer models considered, further suggesting that the CP-PVF distribution may be desirable when considering a mover–stayer random effects distribution for these data. The CP-PVF distribution also contains one less parameter than each of the other mover–stayer random effects distributions considered. Hence, if an information criterion, such as the AIC 25, was to be considered for model selection, then this would provide further evidence in support of the CP-PVF random effects model.

Several authors (e.g. 27–28) have considered zero-inflated Poisson models for count data where there may be a large number of zero counts and devised score tests to determine whether a zero-inflated Poisson model is preferable to a standard Poisson model. This problem is analogous to the problem of a large mass at zero in a mixture model involving a continuous distribution. In addition, Aguirre-Hernández and Farewell 29 considered negative binomial models for the increase in the number of damaged joints exhibited by a patient in the Toronto PsA data set between clinic visits, which allowed for a sub-population of patients who would not exhibit any joint damage. The authors compared a standard negative binomial model with a ‘zero-inflated’ negative binomial model with a larger mass at zero to represent the possibility of a significant sub-population of stayers. They concluded that there was no significant difference in the model fit for the negative binomial model and the zero-inflated negative binomial model. We note that the negative binomial regression models in their study were derived under the assumption of independent gamma random effects acting on the average rate of joint damage between each clinic visit. Solis-Trapala and Farewell 2005 considered a similar zero-inflated negative binomial model for the change in joint count for the Toronto PsA data, only that in this model, patient-level gamma random effects were employed rather than observation-level random effects. Neither of these works found convincing evidence to suggest that a zero-inflated random effects model offered a significant improvement over a standard random effects model. The zero-inflated negative binomial models used corresponded, in each case, to gamma random effects being added to Poisson count models. In light of our work, this lack of evidence to establish a mover–stayer scenario may have been because a gamma random effects distribution was implicitly employed.

The density functions for the gamma and inverse Gaussian mover–stayer models both assume that the parameter representing P(Stayer) *π*, is distinct from the parameters in the distribution function for the movers. This is not the case in the CP-PVF model where the parameter *ρ* governs both the value of P(Stayer) and, to some extent, the shape of the continuous part of the CP-PVF distribution. This feature of the CP-PVF distribution may aid in alleviating problems in estimating the value of P(Stayer) efficiently. We note also that the CP-PVF distribution is the most general of the three random effects distributions considered for these data, having a flexible form that is the sum of independent gamma distributions. It is therefore possible that the likelihood function from this more general model is more informative in this data set.

## 5 Modelling the probability of progression

In the four-state model, a patient who ‘progresses’ through the model is simply one who leaves the initial state, state 1, in at least one joint during his or her time under observation in the clinic. Hence, modelling the probability of progression is equivalent to modelling P(Stayer) which we now consider.

### 5.1 The model

We define *π*_*k*_ to be the probability that the *k*th patient is a stayer (i.e. does not progress) in the four-state model. Suppose that **x**_*k*_ represents a set of patient-level explanatory variables. Then we may model the relationship between *π*_*k*_ and **x**_*k*_ by using a model of the form

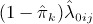

where *g* : [0,1] → ( − ∞, ∞ ) is a suitable link function and ***δ*** represents a vector of regression coefficients representing explanatory variable effects. We will demonstrate how such explanatory variable effects can be incorporated into the four-state model for damage and its relationship with activity, where the mover–stayer inverse Gaussian and CP-PVF random effects were employed. For illustration, we chose to use a patient's erythrocyte sedimentation rate (ESR) at entry to the clinic as the sole explanatory variable in the model for *π*_*k*_. We define the ESR as the rate at which a sample of the patient's red blood cells sediments in 1 hour and it is believed that an increased ESR is a systemic indicator of inflammation. As such, an increase ESR is thought to be associated with an increased level of activity in the joints. Those patients for whom the baseline ESR value was missing had this value replaced using the ESR value from the next available clinic visit, in accordance with standard clinical practice for the imputation of missing ESR values. We note that, of the 510 patients, four patients had no ESR values recorded, and so we omitted these patients from the analysis. In addition, we standardised the baseline ESR values (for each observation, subtracting the sample mean of the baseline ESR values and dividing by the associated sample standard deviation) and employed a complementary log–log link function as *g*(). The complementary log–log link function was particularly convenient for the fitting of the CP-PVF random effects distribution, but in principle, other suitable link functions could be used. Hence, the model for *π*_*k*_ had the form

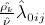

where *x*_*k*_ denotes the standardised baseline ESR value for the *k* patient. We incorporated this into the four-state model, using a CP-PVF distribution, by setting exp( − *ρ*_*k*_) = *π*_*k*_, which implies that *g*(exp( − *ρ*_*k*_)) = *δ*_0_ + *δ*_1_*x*_*k*_. For the mover–stayer inverse Gaussian distribution, the equivalent form is given by *g*(*π*_*k*_) = *δ*_0_ + *δ*_1_*x*_*k*_. We obtained parameter estimates and associated standard errors by using numerical maximum likelihood estimation, as described in Section 2.

### 5.2 Results

In Table 2, we show the results from the fitting of the model where *π*_*k*_ is considered to be a function of baseline ESR. We see that the effects of tenderness, effusion and past activity follow a similar pattern to those estimated for the mover–stayer inverse Gaussian and CP-PVF distributions in Table 1, although for the CP-PVF random effects distribution, the value of each effect is generally reduced when compared with that in Table 1. This may be because the modelling of *π*_*k*_ on baseline ESR has absorbed some of the effects of activity in each patient, seen previously. Where the inverse Gaussian distribution is used, the estimate (95% confidence interval) for *δ*_1_ of − 1.03 ( − 1.93, − 0.14) suggests that the baseline ESR has a significant effect on the probability that an individual is a stayer. Similarly, where a CP-PVF random effects distribution is considered, the estimate (95% confidence interval) for *δ*_1_ of − 0.39 ( − 0.43, − 0.35) also suggests that baseline ESR has a significant effect on the probability that an individual is a stayer. We note that this effect is smaller in magnitude than that where the mover–stayer inverse Gaussian random effects distribution was considered. The negative estimated values of *δ*_1_ for both random effects distributions imply that *π*_*k*_ decreases as the baseline ESR value increases, thereby suggesting that patients with higher baseline ESR values are less likely to have no risk of damage in the hand joints. In clinical practice, the results obtained from fitting this type of model may allow clinicians to target treatment towards those patients for whom they believe that the most intensive treatment is necessary in order to prevent disease progression. We have shown that we can consider the effect of patient-level variables on P(Stayer) where there is reason to believe that a mover–stayer scenario exists within a population, for data that exist as a complicated cluster of several correlated processes.

**Table 2:** Intensity ratio, baseline transition intensity and random effects distribution parameter estimates together with associated 95% confidence intervals fitted to the model incorporating activity and damage to each joint location pair of the left and right hands.

No previous damage in either joint	Intensity ratio		
Effect on transition to damage	M-S inverse Gaussian	CP-PVF
Tenderness in the transitive joint	2.75 (2.05, 3.69)	2.08 (2.05, 2.11)
Effusion in the transitive joint	4.50 (3.41, 5.94)	3.77 (3.69, 3.85)
Activity in the opposite joint	1.17 (0.89, 1.54)	1.02 (1.00, 1.05)
Transitive joint active in the past	2.13 (1.67, 2.70)	1.55 (1.53, 1.56)
Opposite joint active in the past	1.10 (0.86, 1.41)	0.79 (0.76, 0.82)
Opposite joint damaged		
Effect on transition to damage	M-S inverse Gaussian	CP-PVF
Tenderness in the transitive joint	2.22 (1.50, 3.30)	2.07 (1.95, 2.20)
Effusion in the transitive joint	2.17 (1.40, 3.38)	2.20 (2.08, 2.33)
Transitive joint active in the past	1.38 (1.02, 1.89)	0.99 (0.92, 1.07)
Baseline intensities		
Parameter ( × 10^− 2^)	M-S inverse Gaussian *	CP-PVF *
*λ*_012_	0.30 (0.14, 0.64)	0.42 (0.28, 0.56)
*λ*_013_	0.30 (0.14, 0.62)	0.28 (0.17, 0.38)
*λ*_024_	2.29 (0.95, 5.49)	2.46 (0.12, 3.49)
*λ*_034_	2.66 (1.08, 6.56)	1.99 (0.94, 3.03)
Random effect parameters		
Parameter	M-S inverse Gaussian	CP-PVF
*ψ*	0.30 (0.19, 0.48)	
*ν*		182.5 (161.2, 206.6)
Effects of ESR on P(Stayer)	M-S inverse Gaussian	CP-PVF
*δ*_0_	− 1.27 ( − 2.19, − 0.36)	− 0.47 ( − 0.53, − 0.41)
*δ*_1_	− 1.03 ( − 1.93, − 0.14)	− 0.39 ( − 0.43, − 0.35)

We considered the mover–stayer inverse Gaussian and compound Poisson power variance function (CP-PVF) random effects, with the patient-specific probability of non-progression modelled as a function of baseline erythrocyte sedimentation rate (ESR).

* We calculated these estimates and confidence intervals as estimated *expected values* for each distribution, where ESR = 0 for every patient. That is, 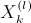 for the M-S inverse Gaussian distribution and 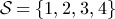 for the CP-PVF distribution.

### 5.3 Testing for the existence of a ‘stayer’ component

Where *π*_*k*_ has the form log( − log(1 − *π*_*k*_)) = *δ*_0_ + *δ*_1_*x*_*k*_, it is clearly not possible to allow *π*_*k*_ = 0, because log(0) is undefined. Moreover, neither *δ*_0_ nor *δ*_1_ exists under the null hypothesis, which is problematic for the formal testing of the existence of a mover–stayer scenario where P(Stayer) depends on an individual-level explanatory variable 31. Matthews and Farewell 1982 have considered similar problems where a specific likelihood ratio test for change-point hazard models was derived, and Matthews *et al.* 1985 discussed asymptotic tests for similar models based on maximal score statistics. Such tests are non-standard, and hence, it is difficult to create a test to compare a mover–stayer model with a non-mover-stayer model where the parameters governing the probability of staying (or moving) depend on explanatory variable values. Pragmatically, it makes little sense to admit a dependence of P(Stayer) on a particular explanatory variable and then test for the existence of a mover–stayer scenario. Instead, it would appear more appropriate to test for a mover–stayer scenario first and then, if supported, consider the regression of P(Stayer) on possible explanatory variables. This strategy would presume that effects of explanatory variables are not very marked. If so, then an analysis of separate subsets of patients may be appropriate.

## 6 Concluding remarks

We have considered the possibility of the existence of two sub-populations of patients within the Toronto PsA study cohort. We define the sub-populations, with regard to damage in the hand joints, as the stayers (those who do not possess the propensity to exhibit damage in any of the hand joints) and the movers (those who do possess the propensity to exhibit damage in one or more of the hand joints). We considered three mover–stayer models to account for the possibility of these sub-populations of movers and stayers. We found that employing a mover–stayer gamma random effect in a four-state multi-state model for the relationship between activity and damage did not produce any demonstrable change from that where a (non-mover-stayer) gamma random effect was used. We considered a mover–stayer inverse Gaussian random effect and produced some evidence to support the idea that a mover–stayer scenario exists within the population.

The use of the chosen CP-PVF random effects distribution in the four-state model has provided the most evidence to suggest that a mover–stayer scenario exists within the population of patients with regard to damage in the hand joints. This represents a novel use of the CP-PVF distribution, in that a form of the distribution has been used both to account for the correlation amongst clustered multi-state processes and for the accommodation of a mover–stayer scenario within the population of patients. We have also shown how the probability of not progressing from the initial state in all joints, P(Stayer), may be modelled as a function of patient-level explanatory variables. We considered a CP-PVF distribution in this scenario and found some evidence that the baseline patient ESR value may be related to a patient's propensity to exhibit joint damage. Here, we performed such modelling as a demonstration, but it could be extended further in future works to include explanatory variables based on genetic information, which some clinicians believe may be important in influencing the onset of damage (e.g. 34).

Although robust guidelines for choosing a suitable mover–stayer multi-state model cannot be given, this work has indicated that a good strategy is to consider more than one possible mover–stayer model. Indeed, if the mover–stayer gamma model alone had been fitted in this work, then a mover–stayer scenario would have been disregarded, thereby conflicting with the evidence accumulated from the fit of the other models considered as well as empirical indications from the data set. One of the most important aspects when considering mover–stayer model is to ask whether such a model is biologically plausible. It may be easy to fit mover–stayer models to many types of time-to-event data and obtain estimates for P(Stayer) (or P(Mover)), but as emphasised by Farewell 1986, it is imperative that any mover–stayer model should be supported by scientific arguments or empirical evidence. It is important to examine the model fitting process carefully to check that the parameters representing the population of stayers are estimable from the data. An examination of the profile likelihood for such parameters is important and should be undertaken. Where possible, a likelihood ratio or score test between a mover–stayer model and a non-mover-stayer model of the same distributional type should be performed in an effort to provide evidence in support of a mover–stayer scenario. Where there are several mover–stayer models, to aid model selection, we can use well-known criteria, such as the AIC.

Overall, there are many possible choices of mixture distribution to model a mover–stayer scenario with regard to a particular event in a data set. We have considered three such distributions for modelling the mover–stayer scenario with regard to hand joint damage in PsA patients. In doing so, it was important to note that different choices of distribution gave widely varying results when estimating the fraction of stayers in the data set. The CP-PVF and mover–stayer inverse Gaussian distributions appear to have been reasonable choices for the random effects distribution here but perhaps not the mover–stayer gamma distribution. Hence, it is important to consider carefully the choice of mixture distribution and pay attention to important distribution features, such as the shape and the parameterisation used (e.g. whether there is any dependence between the shape of the continuous part of the mixture and the size of the point mass at zero or how the density behaves for random effect values close to zero). We recommend that the features of mixture distributions be examined more carefully in future studies where a mover–stayer scenario is suspected, particularly where a fairly complex mover–stayer multi-state model is proposed.

## Appendix A Maximum likelihood estimation for the four-state multi-state model

We define  to be the continuous-time multi-state process for damage at the *l*th joint location for the *k*th patient with state space  and possible transitions given in Figure 1. We write this process as

where  is the time interval of interest with the scale of time used being time since clinic entry in years. Suppose that the baseline transition intensity from state *i* to state *j* at joint location *l* is written as  with , a vector of regression parameters indicating the effects of a vector of individual-specific explanatory variables, , which act upon the state *i* to state *j* baseline transition intensity at time *t*. Assuming that *u*_*k*_ represents the realisation of the random effect, *U*_*k*_, for the *k*th patient, the form of the state *i* to state *j* transition intensity for the *k*th patient at joint location *l* and time *t* is given by

(A.1)

We assume a time-homogenous Markov multi-state model, which implies that the probability of a process existing in state *j* at some time *t*_2_ given that the process is in state *i* at time *t*_1_ (*t*_1_ < *t*_2_) depends on the interval length between the time values *t*_1_ and *t*_2_ rather than the time values themselves, together with the model parameters and relevant explanatory variables. This probability is known as the state *i* to state *j* transition probability and, for the *k*th patient, may be written as *p*_*ijk*_(*t*_1_,*t*_2_ | **z**_*k*_,*u*_*k*_,*ϕ*) where *ϕ* denotes the parameters of the multi-state model (including explanatory variable effects). For the four-state model considered here, one can write the relevant transition probabilities explicitly, and we provide their forms in Section A.1. Suppose that the *k*th patient completes *n*_*k*_ clinic visits, where visit times are represented by , the vector of explanatory variables associated with the *l*th joint location of the *k*th patient at time *t* is denoted by , the baseline transition intensities are denoted by  and the vector of associated parameters for the explanatory variables is denoted by ***β***^(*l*)^. The sequence of states occupied by the *l*th joint location of the *k*th patient, known as the transition path, may be represented diagrammatically as

We calculate the probability of observing the transition path followed by the *l*th joint location multi-state process for the *k*th over the *n*_*k*_ clinic visits, conditional on the random effect, *u*_*k*_, as

The overall contribution to the model likelihood for the *k*th patient, across the 14 joint locations, is given by

Here, , ***β*** = (***β***^(1)*T*^, …, ***β***^(14)*T*^)^*T*^, and  is the probability density function of the random effects distribution. Thus, the likelihood across all *N* patients in the study is given by

(A.2)

We maximised the log-likelihood from Equation 24 with respect to the baseline transition intensity parameters (***λ***_0_), explanatory variable effects (***β***) and random effects distribution parameters (*θ*). We calculated the log-likelihood function by using the statistical program R 15 and performed maximisation of the log-likelihood function by using the BFGS optimisation method (16, implemented using the optim command.

### A.1 Form of the transition probabilities

Suppose that a patient is observed at successive observation times *s*,*t* where *s* < *t*. We provide the analytical form of the transition probabilities *p*_*ijk*_(*s*,*t* | **z**_*k*_,*u*_*k*_,*ϕ*). For convenience, we have dropped the *l* superscript in the equations that follows.

where .

We note that ***β***_*ij*_ is the sub-vector of ***β*** that acts on the transition intensities from state *i* to state *j*. That is, .

## Appendix B The limit of the compound Poisson power variance function probability density function as *U* → 0

The continuous density part of the chosen CP-PVF probability density function (i.e. where *m* = 1) is given by

(B.1)

We aim to prove the statement

We examine the limit of Equation 26 as *u* → 0. Clearly, *f*_*U*_(*u*; *ν*,*ρ*,1) may be written as

(B.2)

Because *ν* > 0, the exponent part of Equation 28 is such that

(B.3)

and hence, the limit of *f*_*U*_(*u*; *ν*,*ρ*,1) as *u* → 0 is determined by the expression

(B.4)

Clearly, all terms of the sum in Equation 30 converge to zero, except the first, as *u* → 0. Thus,

(B.5)

Thus, using the results in Equations 29 and 31, we see that

as required.
